# Prevalence and factors associated with anxiety and depression amongst hospitalised COVID-19 patients in Laquintinie Hospital Douala, Cameroon

**DOI:** 10.1371/journal.pone.0260819

**Published:** 2021-12-02

**Authors:** Stewart Ndutard Ngasa, Leticia Armelle Sani Tchouda, Christabel Abanda, Neh Chang Ngasa, Eric Wah Sanji, Therence Nwana Dingana, Carlson-Sama Babila

**Affiliations:** 1 Medical Research and Careers Organisation, Bamenda, Northwest Region, Cameroon; 2 Manchester University NHS Foundation Trust, Greater Manchester, Manchester, United Kingdom; 3 Faculty of Health Sciences, University of Buea, Buea, Southwest Region, Cameroon; 4 Laquintinie Hospital Douala, Douala, Littoral Region, Cameroon; 5 Tubah District Hospital, Regional Delegation of Public Health, Northwest Region, Cameroon; 6 Nuffield Health Hospitals, The Royal Leamington Spa, Warwickshire, United Kingdom; Konkuk University, REPUBLIC OF KOREA

## Abstract

Studies assessing the mental health of patients with COVID-19 infection remain limited. Disasters and major emergencies, not just COVID-19, undoubtedly lead to greater incidence of mental health problems. Previous studies indicate that the novel Coronavirus disease can cause panic and stress in patients. Our literature search didn’t reveal any previous published data from Cameroon and the Central African sub-region. In order to bridge this gap, we assessed the prevalence and factors associated with depression and anxiety in COVID-19 patients. We carried out a cross-sectional study in a secondary hospital in the Littoral Region of Cameroon. We recruited hospitalised COVID-19 patients during a 4-month period. We collected data on sociodemographic characteristics. The HADS score was used to assess levels of anxiety and depression. All analysis were done using Stata 14. A P value of <0.05 was used as the cut-off for statistical significance. A total number of 285 patients took part in this study with a mean age of 48.47 years. The prevalence of anxiety in COVID-19 patients was 60.35% while the prevalence of depression was 81.40%. At multivariate logistic regression male gender (OR: 1.89, P = 0.04), hypoxaemia (OR: 2.20, P = 0.01), presence of COVID-19 complications (OR: 1.61, P = 0.02) and current episode of depression (OR: 4.14, P<0.01) were independently associated with anxiety. Similarly, age > 35 years (OR:2.03, P = 0.02), presence of comorbidity (OR: 1.68, P = 0.01), BMI > = 30kg/m2 (OR: 1.78, P = 0.02), presence of COVID-19 complications (OR: 1.28, P = 0.01) and anxiety (OR: 4.60, P<0.001) were independently associated with depression. Hospitalised patients with COVID-19 experienced high levels of anxiety and depression. Treatment of hospitalised patients with COVID-19 should therefore include psychotherapy and psychiatric support.

## Introduction

By the end of 2019, a group of patients presenting with signs and symptoms of acute respiratory compromise and idiopathic pneumonia were reported in Wuhan, China. As the cases built up in numbers, the disease was subsequently confirmed to be caused by a novel strain of the Coronavirus and named 2019-nCoV (COVID-19) [1]. Thereon, it rapidly spread throughout the globe; one month after patient zero, the World Health Organisation (WHO) declared it a public health emergency of international concern and 03 months later, it was declared a pandemic [2]. COVID-19 is a life-threatening infectious disease with about 209,201,939 confirmed cases and 4,390,467 deaths recorded globally as of August 2021 [3]. In Cameroon, the first case of COVID-19 was confirmed and isolated on the 06th March 2020 [3] and so far, the country has experienced a steady rise in cases till date; currently 61731 cases and 919 deaths [4].

Though the true impact of COVID-19 cannot be overemphasised, insights regarding the psychological burden beyond the clinical outcomes can also not be ignored as we have learned from prior viral infections with similar pandemic potentials; severe acute respiratory syndrome (SARS) and Middle East respiratory syndrome (MERS) being the more recent examples (2002 and 2012) [5]. Other than the associated considerable morbidity/mortality, prior studies on these highly transmissible infections reported a range of adverse psychological disturbances including but not limited to stress, fear, anxiety, depression, insomnia, sadness, Post-traumatic stress disorder (PTSD) [6,7] and in the worst case scenario, can lead to suicide [8].

Given the overwhelming mortality of COVID-19 (more deaths recorded than from SARS and MERS combined) [9], measures to contain its continual spread such as social and physical distancing (self-isolation, quarantines, lockdowns), travel restrictions, as well as information overload in the media were put in place. These comes with numerous substantial socio-economic and cultural challenges which could trigger or further worsen mental health status. However, most studies assessing the psychological impact of the pandemic have mostly been focused on the general public [10], healthcare workers [11–13], students [14], amongst others.

A few studies have demonstrated high prevalence of anxiety and depression among hospitalised COVID-19 patients. In a study carried out in the United States the prevalence of anxiety and depression among these patients were 34.72% and 28.14% respectively [15]. In a similar study carried out in China, 18.6% of COVID-19 patients were anxious and 13.4% were depressed [16]. While in Nigeria a prevalence of 27.5% and 28.1% were reported for anxiety and depression respectively among COVID-19 patients [17]. Despite these evidence of significant mental distress amongst affected patients, there is a paucity of related data in Cameroon, a country with a growing burden of the disease and a reported low vaccine uptake rates [18] We therefore sort to assess the prevalence of anxiety and depression as well as its associated factors among hospitalised COVID-19 patients at Laquintinie Hospital Douala, Cameroon. This study can reveal valuable depths into the problem and thus help provide a tailored and holistic approach in the management of these patients and hence improve acute outcomes and long-term prognosis.

## Materials and methods

### Study design, setting and participants

We conducted a cross-sectional hospital-based study from April to July 2021 in the Laquintinie Hospital, Douala, Cameroon. The outbreak of COVID-19 let to the construction of isolation units with a capacity of more than 60 beds. These units had 2 doctors and 4 nurses for each shift. Any patient with a confirmed COVID-19 positive test by RDT and/or PCR received a post-test counselling. These patients were then admitted into the isolation units and were treated according to the national protocol for the treatment of COVID-19 as prescribed by the Ministry of Public Health of Cameroon.

### Sampling and data collection

A consecutive convenience sampling technique was used to recruit eligible participants into the study. We pre-tested our questionnaire on a group of 10 patients prior to the commencement of data collection. The patients who took part in the pre-test were excluded from the study. All poorly filled questionnaires were not included in the final analysis.

#### Outcome variables

Our main outcomes were hospital anxiety and depression. These were assessed using the Hospital Anxiety and Depression Scale (HADS). The HADS has been used extensively to measure anxiety and depression in clinical settings. Several review studies have indicated that it has adequate internal, inter-rater, and retest reliability; and convergent, discriminant, and predictive validity [19]. This scale has 14 items with half pertaining to anxiety and half to depression. Each item is scored on a four-point scale ranging from 0 to 3, giving a maximum score of 21 for each subscale. A score of 7 or less is considered normal; scores of 8–10 represent “borderline” and scores of 11 or more indicate the presence of anxiety or depression depending on the relevant subscale [19].

### Independent variables

The following variables were evaluated for association with hospital anxiety and depression: marital status(Single/Divorce, Married), gender (Male, Female), age(years), occupation(Employed and Unemployed), household number, setting (urban or rural), religious denomination (Muslim, Christian, atheists and others); BMI(kg/m2); Number of days of admission; level of education; Presence of comorbidities; Past history of depression and other mental health illnesses; Alcohol consumption; Cigarette smoking; Recent major life event; Presence of COVID-19 complications; COVID-19 Vaccination status; Other family members infected with COVID-19 and Oxygen saturation levels.

### Sample size calculation

The sample size was obtained using the formula for estimation of a proportion since our major outcome was prevalence of hospital anxiety and depression.


$$n=\frac{\left[{4(Zcrit)}^{2}P(1-P)\right]}{D^{2}}$$

Where,

n = Number of participants

Z _crit_ = the standard normal deviation, corresponding to a significance criterion of 0.05 (95), = 1.960

D = Amount of error we will tolerate = ± 6%

P = Pre-study estimate of the prevalence of covid19 vaccine acceptance in health workers = 60%

A pre-estimate value of P = 40% was used. This was in accordance to a similar study in Marrakech, Morocco, where about 40% of hospitalised COVID-19 patients presented with symptoms of depression [20].


$$n=\frac{\left[{4(1.960)}^{2}0.4*0.6\right]}{{\left(0.12\right)}^{2}}$$

n = 256 COVID-19 patients.

### Statistical methods and data analysis

Data were entered into excel spreadsheet and analysed using Stata version 14 statistical software. Results were presented as means and standard deviation (SD) for continuous variables and frequencies and percentages for categorical variables. At bivariate analysis, we used the Cochran-Mantel-Haenszel test to obtain crudes Odd Ratios (OR) of factors associated with hospital anxiety and depression. Multivariate logistic regression was used to identify independent associations with hospital anxiety and depression. This was presented as adjusted odd ratios along with their p-values. A p-value of <0.05 was used as cut off for statistical significance.

### Ethical considerations

We obtained ethical clearance from the Ethical Review Board of the Faculty of Biomedical and Pharmaceutical sciences of the University of Doula prior to commencement of the study. All participants provided verbal and/or written consent before they were recruited into the study.

## Results

### Sociodemographic characteristics of participants

**Fig 1:**
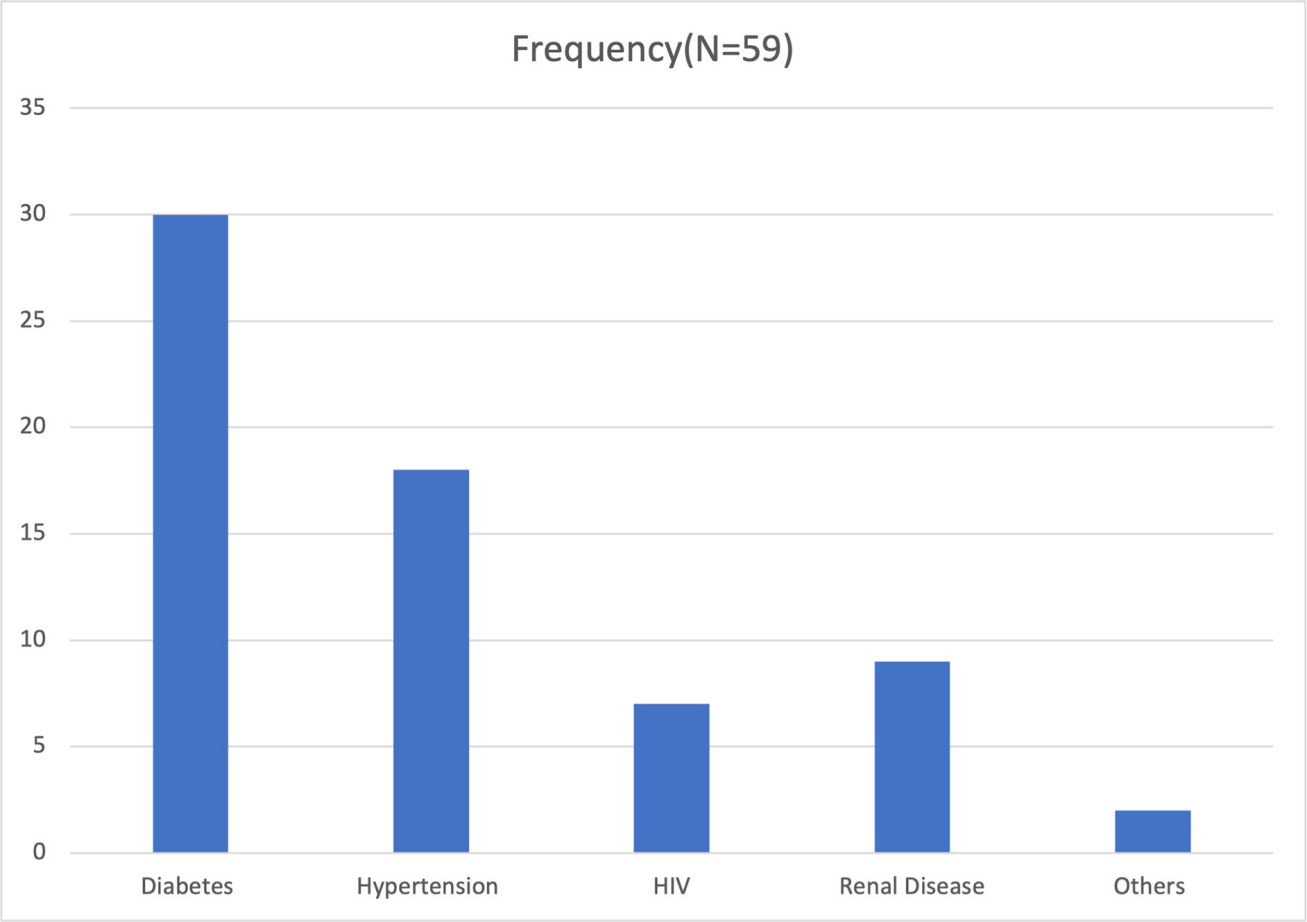
Frequency of comorbidities in study population.

A total number of 285 patients took part in this study with a mean age of 48.47 ±16.01 years and mean BMI of 25.10 ± 4.15kg/m2. The mean number of days of admission for all participants was 5.10 ± 2.52 days. More than two-thirds (67.72%) of participants were male. Majority of the participants were employed (64.56%) and married (62.46%). Almost all participants were Christians (98.25%), without history of other mental health condition (90.53%) and had not received a COVID-19 vaccine (99.30%). The presence of comorbidity was reported by 20.70% of participants (Table 1). Diabetes Mellitus was the most common comorbidity (Fig 1).

**Table 1:** Sociodemographic characteristics of participants.

Variables	Mean ± SD	
**Age(years)**	48.47± 16.01	
**BMI(Kg/m2)**	25.10± 4.15	
**Admission days**	5.10_2.52	
**Oxygen saturation (%)**	80±9.86	
**Anxiety score**	11.36±2.28	
**Depression score**	12.80±2.40	
	**N**	**Percentage (%)**
**Gender**		
Male	193	67.72
Female	92	32.28
**Employment Status**		
Unemployed	101	35.44
Employed	184	64.56
**Education**		
Primary	59	20.70
Secondary	94	32.98
Tertiary	132	46.32
**Marital Status**		
Single/Divorced	107	37.48
Married	178	62.46
**Religion**		
Muslim	5	1.75
Christians	280	98.25
Others	0	0
**Presence of comorbidity**		
Present	59	20.70
Absent	226	79.30
**History of depression**		
Yes	56	19.65
No	229	80.35
**History of other mental health conditions**		
Yes	27	9.47
No	258	90.53
**Consumes alcohol**		
Yes	257	90.18
No	28	9.82
**Vaccination status**		
Vaccinated	2	0.70
Not vaccinated	283	99.30
**Infected family members**		
**Yes**	54	18.95
**No**	231	81.05

### Prevalence of anxiety and depression

**Fig 2:**
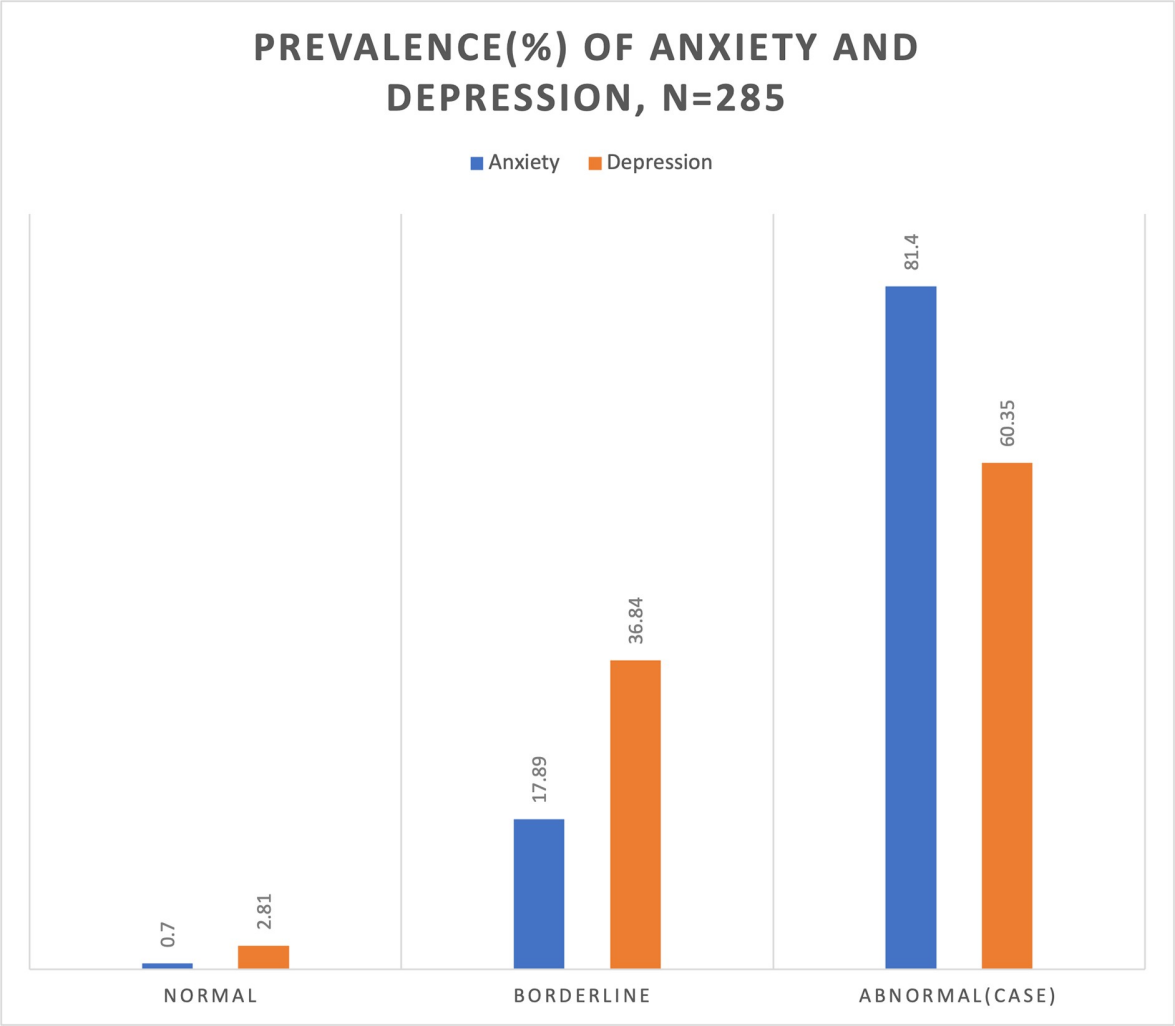
Prevalence of depression and anxiety among hospitalised COVID-19 patients.

The mean anxiety and depression scores were 11.36±2.28 and 12.80±2.40 respectively. The prevalence of anxiety in COVID-19 patients was 60.35% while the prevalence of depression was 81.40% (Fig 2).

### Factors associated with depression

At bivariate analysis, age > 35 years (OR:2.56, P = 0.002), presence of comorbidity (OR:1.59, P<001), obesity (OR: 2.70, P<0.001), being employed (OR:1.83, P = 0.04), hypoxaemia (OR:2.94, 0.002), presence of COVID-19 complications (OR: 1.72, P = 0.001) and anxiety (OR: 5.96, P<0.001) were associated to depression (Table 2).

**Table 2:** Factors associated with depression.

Variables		Crude ORs	P-value	Adjusted OR	P-value
Marital status	Married	1.31	0.25	*	*
	Single/Divorce	1			
Gender	Male	1.64	0.10	1.0	0.96
	Female	1			
Age group	>35	2.56	0.002	2.03	0.02
	< = 35	1			
Presence of comorbidity	Yes	1.59	<0.001	1.68	0.01
	No	1			
BMI	Obesity (= >30kg/m2)	2.70	<0.001	1.78	0.002
	< 30kg/m2	1			
Level of education	University	1.31	0.07	*	*
	Primary/Secondary	1			
Employment status	Employed	1.83	0.04	1.20	0.60
	Unemployed	1			
Days of hospitalisation	> = 5 days	1.29	0.53	*	*
	< 5 days	1			
Oxygen saturation	Hypoxaemia (< 80)	2.94	0.002	2.14	0.07
	> = 80	1		1	
Alcohol consumption	Yes	1.30	0.54	1.01	0.90
	No	1			
Smoking	Yes	1.24	0.49	*	*
	No	1			
History of mental health condition	Yes	1.92	0.20	*	*
	No	1			
History of depression	Yes	1.60	0.08	*	*
	No	1			
Presence of COVID-19 complications	Yes	1.72	0.001	1.28	0.013
	No	1		1	
Presence of family support	Yes	1.49	0.25	*	*
	No	1			
Anxiety	Yes	5.95	<0.001	4.60	<0.001
	No	1		1	

*Excluded in the multivariate logistic regression model.

The following variables were independently associated with depression: age > 35 years (OR:2.03, P = 0.02), presence of comorbidity (OR: 1.68, P = 0.01), BMI > = 30kg/m2 (OR: 1.78, P = 0.02), presence of COVID-19 complications (OR: 1.28, P = 0.01) and anxiety (OR: 4.60, P<0.001) (Table 2).

### Factors associated with anxiety

Crudes associates of anxiety identified in this study included, being married (OR: 1.55, P = 0.02), being a male patient (OR: 2.29, P = 0.001), age > 35 (OR: 2.59, P = 0.003), obtaining at least a university education (OR: 1.28, P = 0.04), hypoxaemia (OR:2.89, P<0.001), history of depression (OR: 1.63, P = 0.001), presence of COVI-19 complications (OR:1.73, P<0.001) and current depressive episode (OR: 5.96, P<0.001) (Table 3).

**Table 3:** Factors associated with anxiety.

Variables		Crude ORs	P-value	Adjusted OR	P-value
Marital status	Married	1.55	0.02	1.43	0.11
	Single/Divorce				
Gender	Male	2.29	0.001	1.89	0.04
	Female				
Age group	>35	2.59	0.003	1.48	0.23
	< = 35	1			
Presence of comorbidity	Yes	1.1	0.86	*	*
	No				
BMI	Obesity (BMI > 35)	1.29	0.29	*	*
	BMI < 35kg/m2	1			
Education	University	1.28	0.04	1.16	0.40
	Primary/Secondary	1			
Employment status	Employed	1.65	0.04	1.30	0.40
	Unemployed				
Days of hospitalisation	> = 5 days	1.16	0.53	*	*
	< 5 days	1			
Oxygen saturation	< 80	2.89	<0.001	2.20	0.01
	> = 80	1			
Alcohol consumption	Yes	1.87	0.11	*	*
	No				
Smoker	Yes	1.09	0.78	*	*
	No				
History of mental health condition	Yes	2.40	0.05	*	*
	No				
History of depression	Yes	1.63	0.001	1.49	0.20
	No				
Presence of Life event	Yes	1.33	0.40	*	*
Presence of COVID-19 complications	Yes	1.73	<0.001	1.61	0.02
	No				
Presence of family support	Yes	0.60	0.10	*	*
	No				
Depression	Yes	5.95	<0.001	4.14	<0.001
	No				

*Excluded in the multivariate logistic regression model.

At multivariate logistic regression male gender (OR: 1.89, P = 0.04), hypoxaemia (OR: 2.20, P = 0.01), presence of COVID-19 complications (OR: 1.61, P = 0.02) and current episode of depression (OR: 4.14, P<0.01) were independently associated with anxiety (Table 3).

## Discussion

Given the increasing rates/burden of mental illnesses globally, the need to assess the psychological impact of COVID-19 is crucial; particularly amongst hospitalised patients of black origin who disproportionately suffer more adverse outcomes than other demographics [21,22]. Such outcomes maybe further compounded in resource-limited settings where the burden of unmet psychological needs is higher. However, data defining the scale of the problem in these settings is scarce as reflected in a recent meta-analysis [23] wherein study findings were limited by lack of data within the African region. It is with this backdrop that we assessed the level of anxiety and depression as well as their associated factors in COVID-19 patients admitted at a second category hospital in Cameroon. Our study demonstrated that about two-thirds of patients (60.35%) presented with anxiety while more than 80% of them presented with depression. These results were two to four times higher than those previously reported in other cross-sectional studies [15–17]. Our findings also mirror that of a systematic review conducted by Wu et al [24] which reported a high prevalence of a range of mental health problems (anxiety and depression inclusive) resulting from the COVID-19 pandemic [24].

In an online survey of COVID-19 patients in Nigeria, 27.5% and 28.1% of respondents presented with anxiety and depression respectively [17]. The lower prevalence of anxiety and depression reported in their study could be due to the fact that they recruited everyone who had tested positive for COVID-19. In our study we included only hospitalised patients with severe COVID-19 infections. Having a cohort of sicker patients in our study might have let to the increase level of anxiety and depression. In a similar study in Wuhan, 18.6% of participants experienced anxiety symptoms while 13.4% experienced depressive symptoms [16]. The Wuhan study had a smaller study population and was carried earlier on during the pandemic, when there was limited information on the lethality of the virus.

Despite the varying methodological approaches employed by these studies and limitations faced, the overall evidence still suggests that the pooled prevalence of psychological disturbances (anxiety, depression, stress, sleep problems) during the COVID-19 pandemic is significantly higher than pre-pandemic levels [23]. It is also worth mentioning that such high levels of psychological disturbance have been shown to persist beyond the acute phase with some studies finding up to 35% of patients with residual anxiety and depression months post-hospitalisation with a virus of similar pandemic potentials [25]. Thus, if trends were to continue, the attention this deserves from the relevant authorities cannot be underestimated.

A number of factors have been shown to be associated with anxiety and depression. Independent risk factors for anxiety identified in our study included: hypoxaemia, male gender, presence of COVID-19 complications, and comorbid depression. These findings have been corroborated in previous studies where it has been demonstrated patients with low oxygen saturation levels were more likely to be anxious [20,26]. It has been postulated that the increase anxiety might not just be linked to the physical stress of the illness but also to the side effects of the treatment given to patients [19]. We also found that male patients were at higher risk of anxiety. This finding contradicts those of previous studies which demonstrated that females were more likely to develop mood disorders and stress-related mental illness including depression and anxiety [15,20]. Finally, the observed association of high levels of anxiety with COVID-19 complications and comorbid depression has been demonstrated in a study by Mazza et al. [27]. In their study, it was postulated that increase levels anxiety and depression as a long-term sequela of COVID-19 infection could be explained by the inflammatory changes cause by the infection.

Independent risk factors for depression included: age > 35 years, presence of comorbidity, obese patients, the presence of COVID-19 complications and comorbid anxiety. It has been shown that older patients were more likely to be depressed [20]. Also, another study revealed that the presence of other comorbidity was associated with higher levels of depression [17]. Our study also showed a strong evidence of an association between obesity and depression in COVID-19 patients. Obesity is on the rise, it is now a second pandemic running parallel to the COVID-19 pandemic [28]. It has been demonstrated that obesity has a strong positive correlation with depression leading to fatal outcomes in COVID-19 patients [29]. Physical activity is beneficial to mental health and immunity. Encouraging and helping individuals to be physically active during the COVID-19 pandemic could reduce the risk of depression and other mental health problems. Anxiety and depression usually co-exist in many people suffering from mental health conditions. This co-existence has been demonstrated in patients with COVID-19 infection during the acute infection(as demonstrated in our study), and as a long term sequelae of the disease [27].

### Strengths and limitations

There were several strengths to this study: to the best of our knowledge, this was the first study to assess levels of anxiety and depression amongst hospitalised COVID-19 patients in Cameroon. We also included a larger sample size compared to the previous studies we identified in the literature search. Finally, a consecutive random sampling method was used to recruit patients into the study. This helped minimize selection bias in our study.

There were however some limitations to our study:

We recruited patients from a single COVID-19 treatment centre in Cameroon. Thus, it might be misleading to generalise the findings to the entire country or sub-region. Also, most of the data were self-reported by the recruited patients, some of them in distress at the time of data collection. This can lead to high level of recall bias. Further, the snapshot study design implies causality cannot be inferred.

Regardless, being a pioneer study within the country, the findings have implications for healthcare providers and policy makers as it underlines the high rates of mental health issues among hospitalised COVID-19 patients. Furthermore, not only does the data sets the foundation for future studies; it also underpins the importance of a holistic approach in managing these patients, taking into account both the clinical and phycological aspects. This might be invaluable in the prevention of adverse short- and long-term outcomes.

## Conclusion

The results demonstrated that hospitalised patients with COVID-19 experienced high levels anxiety and depression. Anxiety was associated with male gender, hypoxaemia, presence of COVID-19 complications and comorbid depression while depression was associated with age > 35 years, presence of comorbidity, obesity, the presence of COVID-19 complications and comorbid anxiety. Therefore, psychological and psychiatric support should be an integral part in the management of these patients.

## Supporting information

S1 File (XLSM)
